# Musical rhythm effects on visual attention are non-rhythmical: evidence against metrical entrainment

**DOI:** 10.1093/scan/nsaa077

**Published:** 2020-06-08

**Authors:** Annett Schirmer, Maria Wijaya, Man Hey Chiu, Burkhard Maess, Thomas C Gunter

**Affiliations:** Department of Psychology, The Chinese University of Hong Kong, Shatin, Hong Kong SAR; The Brain and Mind Institute, The Chinese University of Hong Kong, Shatin, Hong Kong SAR; Department of Psychology, The Chinese University of Hong Kong, Shatin, Hong Kong SAR; Department of Psychology, The Chinese University of Hong Kong, Shatin, Hong Kong SAR; Max Planck Institute for Human Cognitive and Brain Sciences, Leipzig 04103, Germany; Max Planck Institute for Human Cognitive and Brain Sciences, Leipzig 04103, Germany

**Keywords:** entrainment, foreperiod, EEG, music, tapping, timing, frequency tagging

## Abstract

The idea that external rhythms synchronize attention cross-modally has attracted much interest and scientific inquiry. Yet, whether associated attentional modulations are indeed rhythmical in that they spring from and map onto an underlying meter has not been clearly established. Here we tested this idea while addressing the shortcomings of previous work associated with confounding (i) metricality and regularity, (ii) rhythmic and temporal expectations or (iii) global and local temporal effects. We designed sound sequences that varied orthogonally (high/low) in metricality and regularity and presented them as task-irrelevant auditory background in four separate blocks. The participants’ task was to detect rare visual targets occurring at a silent metrically aligned or misaligned temporal position. We found that target timing was irrelevant for reaction times and visual event-related potentials. High background regularity and to a lesser extent metricality facilitated target processing across metrically aligned and misaligned positions. Additionally, high regularity modulated auditory background frequencies in the EEG recorded over occipital cortex. We conclude that external rhythms, rather than synchronizing attention cross-modally, confer general, nontemporal benefits. Their predictability conserves processing resources that then benefit stimulus representations in other modalities.

## Introduction

Musical rhythms powerfully influence human listeners by making them tap, sway or dance in synchrony. This, often involuntary, response is thought to be part of a more general entrainment process including, for example, the rhythmical alignment of cross-modal attentional oscillations. Here we investigated a rhythm’s influence on attention by testing the role of metricality, the key defining feature of musical rhythms, and pitching that against regular temporal that characterize both rhythmical and non-rhythmical sound streams.

**Fig. 1:**
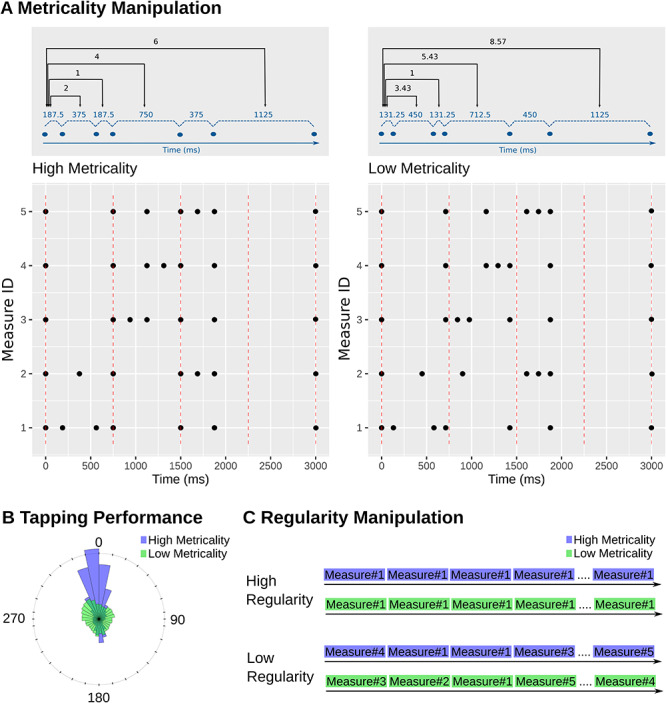
Stimulus background manipulation. (A) Metrical properties of the measures used to create the background rhythms. The upper part of panel A illustrates one exemplary rhythm in its high (left) and low (right) metricality variant. Shown in blue are the within-measure intervals in milliseconds. Shown in black is the ratio of each interval to the smallest interval. The lower part of the figure illustrates the temporal position of sounds for all the measures in the experiment. The red dashed lines mark the position of the beat. (B) Histogram of tapping performance illustrated as a roseplot. Rose paddles represent cumulative participant taps for the five high (violet) and the five low metrical measures (green). Larger paddles reflect more taps at a given angle. The 0 position reflects the beat which occurred every 750 ms. (C) Regularity manipulation. Metricality and regularity were orthogonally manipulated. In the high regular condition, one of the high metrical or the low metrical measures was repeated throughout a block. In the low regular condition, we presented a random order of all high or all low metrical measures.

The idea that musical rhythms entrain the human mind was first formalized in Jones’ dynamic attending theory (DAT); (Jones, 1976; Jones and Boltz, 1989). In this theory, Jones argued that mental processes like attention are not consistent but oscillate between performance peaks and troughs. She also developed a temporal perception account that diverged from classical frameworks. Jones suggested that, instead of relying strictly on isolated intervals or durations, we also leverage on temporal structures afforded by the environment. If these structures are metrical in that intervals are nested hierarchically resulting in integer ratios (Figure 1), they facilitate the development of rhythmic expectations and the entrainment of attention such that attentional peaks align with salient temporal positions. Please note that although metricality is typically treated as a binary phenomenon, whether it is indeed binary is questionable. One might speculate that ratios approaching an integer are more metrical than ratios further way from an integer, and to accommodate this possibility, we here treat metricality as a continuous construct.

Since its inception, DAT received much empirical support and has been instrumental in the linking of oscillations in behavior and brain function (e.g. Buschman and Kastner, 2015). Looking specifically at attention, Jones and colleagues devised a now popular paradigm that we will present in a bit more detail here (Jones *et al.,* 2002). In its original form, participants listened to two tones separated by a retention interval and decided whether the final comparison tone had the same, a higher or lower pitch than the first tone. Distractor tones varying in pitch were presented during the retention interval isochronously, that is, with a regular stimulus-onset asynchrony (SOA). The final comparison tone could occur with that same SOA, slightly earlier or slightly later. Participants responded more accurately when the target temporally preserved rather than violated the isochronous sequence. Moreover, this was also true when the target occurred at twice the SOA, implying that listeners represented and used the underlying meter.

With this and similar paradigms, much evidence accumulated showing that humans are sensitive to and benefit from the temporal structure of stimulus streams not only in the auditory (Chang *et al.,* 2019; Bouwer *et al.,* 2020) but also in the visual (Rohenkohl and Nobre, 2011; Cravo *et al.,* 2013) and tactile modality (Jones *et al.,* 2017; Jones, 2019). Moreover, the effect of rhythm was shown to transcend modalities, leading to the entrainment of modality-unspecific attending. In one of the first attempts to demonstrate this, McAuley and colleagues presented a central fixation point that, after a waiting interval, moved into one of the screen’s corners (Miller *et al.,* 2013). The waiting interval was filled with tones spaced isochronously, and the following change in fixation point location could occur with an identical SOA as to preserve the intervening rhythm or slightly earlier or later. Results replicated unimodal findings in that synchronous visual change was more readily tracked as indexed by a shorter saccade latency. Moreover, saccade differences between the different SOA conditions disappeared when the auditory sequence was randomly timed. Other research has corroborated these results and provided further support for DAT (Escoffier *et al.,* 2010, 2015; Brochard *et al.,* 2013; Bolger *et al.,* 2014; Trost *et al.,* 2014; Johndro *et al.,* 2019).

Although seemingly intuitive, the study of rhythmic timing and its impact on associated mental processes is quite challenging. Moreover, existing work is compromised by one or more of the following three methodological shortcomings limiting conclusions about entrainment.

First, the traditional entrainment paradigm as described above confounds rhythmic or beat-based with stimulus expectations. Specifically, the metrical structure of rhythms leads to the perception of a regularly spaced temporal emphasis called the beat. For an isochronous sequence, the most simple kind of rhythm, beat perception, coincides with each stimulus in the sequence. However, for nonisochronous and more complex rhythms, beats may be perceived at points that are not acoustically marked (e.g. see high-metrical examples in Figure 1). By relying on isochronous sequences, most existing studies, hence, tested attention at points in time where participants expected both a beat and a stimulus rather than just a beat. To address this point, some attempts have been made to employ more complex rhythms that develop expectations for a silent beat at which a target may then be presented (Escoffier *et al.,* 2010; Tal *et al.,* 2017).

A second problem concerns the dissociation between the metricality and regularity of a stimulus sequence (Obleser *et al.,* 2017). As mentioned above, metricality arises from stimulus sequences in which smaller intervals form an integer ratio with larger intervals. Perhaps because such sequences create the perception of a regularly spaced beat, they are often also referred to as regular. Yet, upon careful consideration one may wish to dissociate metricality and regularity. Again in the simplest, isochronous case, the same interval is being repeated over and over again inducing a beat and maximizing interval regularity. In a more complex, nonisochronous case, however, intervals varying within and across measures may still induce a beat despite being a lot less regular. Thus, the traditional comparison of an isochronous condition with a condition in which successive intervals vary randomly with non-integer ratios confounds metricality with regularity. It leaves open whether metricality and its hierarchical organization of time is critical for temporally aligning mental processes. Alternatively, the brain may simply rely on any regularity in the environment irrespective of its structure (Friston, 2010; Breska and Deouell, 2017; Rimmele *et al.,* 2018; Herbst and Obleser, 2019).

Last, a great challenge has been to control the influence of local temporal cues on timed behaviors. The final stimulus of an entrainment sequence presented prior to the target can be considered a warning signal and the delay between this stimulus and the target can be considered a foreperiod. Much research has shown that foreperiod duration impacts reaction times as a function of other experimental parameters. For example, when foreperiods vary within a block, longer foreperiods are associated with faster responses. However, the opposite is true when foreperiods are held constant up to a threshold of about 70 ms (Niemi and Näätänen, 1981). Additionally, the salience of the warning stimulus, the distribution of experienced foreperiods and whether targets are occasionally omitted (Polzella *et al.,* 1989) are all relevant. In fact, depending on the context, the foreperiod may start even before the warning signal or the last stimulus before the target (Ellis and Jones, 2010). As such presenting a target synchronously or asynchronously with a rhythm has complex consequences for local cue-based preparatory processes.

Although attempts have been made to address one or more of the aforementioned issues (e.g. Escoffier *et al.,* 2010; Rohenkohl *et al.,* 2012; Cravo *et al.,* 2013; Miller *et al.,* 2013; Herrmann *et al.,* 2016; Tal *et al.,* 2017), so far they have not been considered together, thus leaving us without a strong test of the assumptions of DAT in general and cross-modal rhythmical entrainment in particular. Here we aimed at providing such a test. Specifically, we asked participants to monitor the color of a fixation cross while passively listening to an auditory sequence. The temporal structure of this sequence varied orthogonally in metricality and regularity across different stimulus blocks. Metricality was manipulated via the ratio of intervals within measures making up a sequence. Regularity was manipulated by either varying or repeating the measures within a sequence. Irrespective of metricality and regularity, all sequences comprised a silent period including the fourth beat in the highly metrical condition. Additionally, the number of notes and the silent period duration were held constant. As in previous studies (Escoffier *et al.,* 2010, 2015; Brochard *et al.,* 2013; Tal *et al.,* 2017), we manipulated the target’s temporal position by presenting task-relevant color changes either on the silent beat or slightly earlier or later. However, rather than comparing the different target positions within one background condition, we examined the background effect on one target position. This latter approach enabled us to control local temporal processes associated with different foreperiods.

If indeed auditory stimuli entrain attention as suggested by DAT and such entrainment emerges cross-modally, we should observe an interaction between a target’s temporal position (i.e. a/synchrony with the silent beat) and background metricality. Moreover, synchronous targets should be processed more efficiently when metricality is high as compared to low. A similar effect should be absent for non-synchronous targets. Alternatively, auditory stimuli may modulate visual attention as a function of regularity. If so, targets should be processed more efficiently when regularity is high as compared with low, and this effect should be independent of the targets’ temporal position.

## Methods

### Participants

We recruited 75 participants for the main experiment and of those 11 were excluded from data analysis due to experimenter error (*N* = 8), flat channels during recording (*N* = 2) or missing data (*N* = 1). Half of the remaining 64 participants were female with an average age of 24 years (s.d. 4), and the other half were male with an average age of 24 years (s.d. 3). All female participants were right-handed. Twenty-nine male participants were right-handed, one was ambidextrous, and for two handedness was unavailable.

### Stimuli

Our background rhythms were designed based on 10 especially composed measures. Half of these measures were high, and the other half were low in metricality. High metricality measures induced four beats whereby the first three beats were acoustically marked and the fourth beat was silent. High metricality measures served as the basis for the composition of low metricality measures. These latter measures had the same duration and comprised the same number of sounds as their originals. However, sounds were shifted to fall on off-beat positions with the exception of the first and last note in the measure. Moreover, whereas the intervals in the high-metrical rhythms were related by ratios of 1:2:4:6, they were related by ratios of 1:3.43:5.43:8.57 in the low metrical rhythms. Figure 1A illustrates sound timings for each of the 10 measures.

We tested the metricality manipulation in a separate tapping experiment in which 16 individuals (8 female, mean age = 26 years), not participating in the main experiment, were asked to tap along each of the measures described above. Each measure was looped 40 times in a separate block. The five metrical and the five nonmetrical blocks were always presented consecutively forming two supra-blocks. The order of blocks within each supra-block was randomized for each participant. Participants were asked to close their eyes and tap using their dominant hand. Their taps were recorded on a custom-built touch pad with a temporal resolution of 1 ms. We analyzed the tapping data in a circular statistics framework. This means that instead of treating time linearly, we conceptualized the 750 ms interval between beats to form a 360° circle with the silent beat in the 0 position and the delay between this position and the participants’ tap expressed as an angle. A Rayleigh test indicated that taps were nonuniformly distributed around the beat when metricality was high (statistic = 0.746, *P* < 0.0001) but not when it was low (statistic = 0.178, *P* = 0.159). Additionally, the vector length associated with the mean tapping angle was significantly greater in the high (0.41) as compared with the low metricality condition (0.12; *F*[1,15] = 15.48, *P* < 0.0001, η_G_^2^ = 0.278). Thus, together both tests demonstrate that, as expected, high metricality enabled good beat alignment and regular tapping. By contrast, there was no evidence for the measures with low metricality that participants tapped selectively on the position coinciding with the beat in high-metrical measures. Additionally, their tapping was significantly more variable (Figure 1B).

During the experiment, auditory sequences were played at comfortable loudness (~65 dB) using two loud speakers positioned to the left and right of the monitor.

### Procedure

**Fig. 2:**
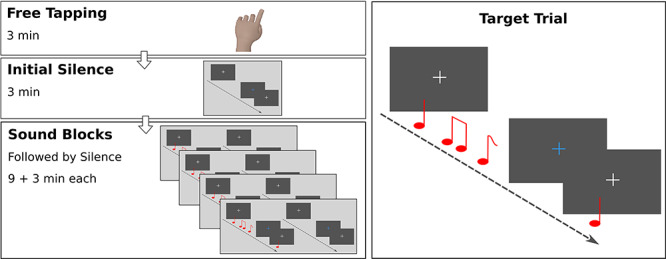
Primary experiment procedure. Illustrated on the left is the blocked outline of the experiment. A free tapping exercise was used to measure spontaneous tempo. This was followed by a 3 min period in which participants performed the visual target detection task in silence. Afterwards, participants were presented with four blocks each comprised of a 9 min sound and 3 min silence phase. Illustrated on the right is a target trial. For half the measures/trials, the fixation cross changed color for 100 ms prompting participants to press a button.

The experiment was divided into several phases (Figure 2). First, participants were asked to tap with their dominant hand on a custom-built touch pad for 3 min. Subsequently, they completed a visual target detection task in silence. This task block is referred as ‘initial silence’ and was followed by four more visual target detection blocks. During the initial larger part of these blocks, one of the auditory conditions played in the background (high metrical/high regular, high metrical/low regular, low metrical/high regular, low metrical/low regular). The final shorter part of each block was silent. Both block parts are referred to as sound and post-sound silence. In what follows, we will explain each study phase in greater detail. We begin by describing the visual target detection task and then proceed to explaining how this task was combined with sound background and silence. At the end of this section, we will shortly integrate the various procedural elements.

During the visual target detection task, participants were asked to focus on a white fixation cross presented on a gray background and to press a button in case the cross changed intermittently to blue. Changes in fixation cross color lasted for 100 ms. The onset time of color changes varied. This variation was designed based on the temporal properties of the auditory background measures and was comparable across the different experimental blocks. Because our auditory stimulus measures served as the basis for target-to-target intervals, we will first explain the timing of targets when there was auditory background and then detail the timing of targets when there was silence.

**Fig. 3:**
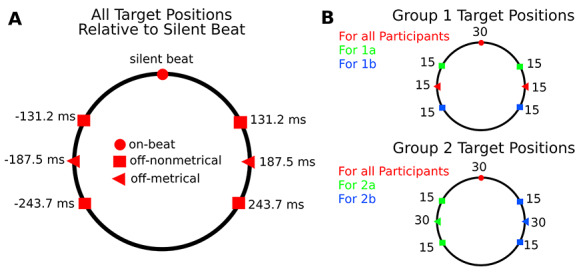
Target positions in the experiment. (A) Illustrated on the left are all possible target positions relative to the onset of the silent beat. The different shapes illustrate on-beat, metrical off-beat and nonmetrical off-beat positions. (B) Illustrated on the right are the distributions of possible target positions as a function of participant group. The different colors index which group saw which target position and the numbers indicate how often that target position was presented. Again, the different shapes illustrate on-beat, metrical off-beat and nonmetrical off-beat positions. For group 1, the beat position was central among target positions, whereas in group 2, the beat position was peripheral. This allowed us to examine the role of foreperiod distribution and a potential benefit resulting from a central beat position.

The measures used for the different sound conditions all shared a silent period of 1125 ms surrounding the forth beat position. Specifically, the last note of a given measure played 375 ms after the third beat position, and the next note was the first note of the subsequent measure. Targets could be aligned with the silent beat position, with an earlier or later metrical off-beat position or an earlier or later nonmetrical off-beat position (Figure 3). Note that for ease of reference, we will refer to these positions as on-beat, metrical off-beat and nonmetrical off-beat positions both in the context of high and a low metricality backgrounds. Metrical off-beat positions fell 187.5 ms before and after the fourth silent beat position, which represents the beats quarter subdivision. To match the metrical off-beat positions’ average temporal distance from the beat, nonmetrical off-beat positions occurred equidistantly either 56.3 ms before or after each metrical off-beat position. Please see Figure 3A for a graphical illustration of possible presentation time points.

Each participant experienced 30 on-beat, 30 metrical off-beat and 30 nonmetrical off-beat targets across 180 measures in each sound phase. Target time points as well as measures with and without targets were presented in random order. In an effort to explore and control for foreperiod distribution effects, not all participants were exposed to all metrical and nonmetrical off-beat time points. As illustrated in Figure 3B, participants were divided into four groups (1a, 1b, 2a, 2b) with different time point combinations. Groups 1a and 1b had 30 on-beat targets and 30 metrical off-beat targets (15 before and 15 after the beat). Additionally, group 1a had 30 nonmetrical off-beat targets closer to the beat (15 before/15 after), whereas group 1b had 30 nonmetrical off-beat targets further away from the beat (15 before/15 after). Groups 2a and 2b had 30 on-beat targets. Additionally, group 2a had 30 metrical off-beat targets before the beat and 30 nonmetrical off-beat targets around this early metrical off-beat position (15 before/15 after). Group 2b had 30 metrical off-beat targets after the beat and 30 nonmetrical off-beat targets around this late metrical off-beat position (15 before/15 after). Our primary analysis reported below was done across all four groups, which controls for foreperiod distribution. The interested reader can find an analysis of foreperiod distribution effects in the Supplementary Materials.

As described earlier, metricality was manipulated by adjusting the intervals within a measure and regularity by adjusting the number of times a measure was repeated within a background block. This resulted in four sound background conditions: (i) the high-metrical and high-regular condition simply looped one of the five high-metrical measures throughout the block, (ii) the high-metrical and low-regular condition presented all five high-metrical measures in random order, (iii) the low-metrical and high-regular condition looped one of the five low-metrical measures throughout the block, and (iv) the low-metrical and low-regular condition presented all five low-metrical measures in random order. Across participants, all high and low metricality measures occurred equally often in the regular conditions with the exception of measure 1 which occurred 1 more time each for male and female participants due to the counterbalancing constraints described further below.

The time points developed for target presentation were held constant across sound and silence phases. During the initial silence phase and the post-sound silence, participants were presented with 30 targets with 10 each falling on on-beat, off-metrical and off-nonmetrical positions, respectively. These positions had no meaning in the context of silence but simply preserved the range of target-to-target intervals across sound and silence.

In sum, an experimental session began with a tapping exercise (see Supplementary Materials for results). Subsequently, participants completed the experimental task in silence followed by four additional task blocks each comprised of a sound and post-sound silence phase. The order of these latter four blocks was counterbalanced by nesting the factor metricality in regularity and vice versa and by presenting the superordinate and nested levels in all possible orders, resulting in a total of 32 counterbalancing groups. Each sound phase took 9 min in which 180 measures were played consecutively. The initial and post-sound silence phases took 3 min each and were followed by a short break.

### Electrophysiological recording and analysis

The EEG was recorded using 61 Ag/AgCl electrodes, which were located according to the extended 10–20 system of the American Clinical Neurophysiology Society (2006). An additional four electrodes were attached above and below the right eye and at the outer canthus of each eye to measure eye movements. The left mastoid was used as online reference. Electrode impedance was below 5 kΩ. The data was recorded at 500 Hz with a BrainAmp EEG system. Only an anti-aliasing filter was applied during data acquisition (i.e. sinc filter with a half-power cut-off at half the sampling rate).

EEG data were preprocessed with EEGLAB v14.1.1 (Delorme and Makeig, 2004). The recordings were high-pass filtered with a −6 dB cut-off at 0.1 Hz (0.2 Hz transition bandwidth, zero-phase FIR, 8251 points). For the ERP analysis, the continuous data were scanned visually to remove nontypical artifacts caused by muscle movements, poor connections or electrode drifts. Channels with excessive artifacts were interpolated. Afterwards, the data were re-referenced to the scalp average. A 0.5 Hz high-pass filter (1 Hz transition bandwidth, zero-phase FIR, 1651 points) was applied prior to subjecting the data to adaptive mixture-independent component analysis (AMICA) (Palmer *et al.,* 2011). The resulting independent component structure was applied to the original data with the 0.1 Hz filter setting. Components reflecting typical artifacts (i.e. horizontal and vertical eye movements and eye blinks) were removed and the data back-projected from component space into EEG channels space. Another visual scan was done to remove data with residual artifacts, a low-pass filter was applied at 30 Hz (7.5 Hz transition bandwidth, zero-phase FIR, 221 points), and the signal was epoched separately for measure and target onset using a time window from −200 to 500 ms around measure and target onset. Bad epochs were rejected automatically using the joint probability and thresholding functions in EEGLAB with the rejection criteria set at three s.d. and ±100 μV, respectively. The epoched data were submitted to a current source density transformation using the CSD tool box (Kayser and Tenke, 2006) with its default settings and baseline corrected using the 200 ms pre-stimulus period.

For the analysis of measure onset ERPs, the number of correct/artifact-free trials ranged from 125 to 179 with a mean of 164 for each combination of metricality and regularity. For the analysis of target onsets, we separately considered the initial silence, sound and post-sound silence phases. The initial silence phase had, after artifact rejection, trial numbers ranging from 3 to 10 with a mean of 9 for on-beat, metrical off-beat and nonmetrical off-beat positions, respectively. Ideally we would have confirmed the absence of ERP differences between these positions. Due to the low trial numbers, however, this was not done. The post-sound silence had, for each sound condition, a minimum of 10, a maximum of 30 and a mean of 27 trials after artifact rejection. We conducted separate ANOVAs with trial number as the dependent variable for the sound and post-sound silence phases and found that trial numbers did not differ significantly between conditions (all *P*s > 0.102). The factors used in these ANOVAs were identical to those used in the ERP analysis reported below. Prior to the analysis of target onset ERPs, the average of nontarget (control) epochs was subtracted from the average of target epochs to (i) eliminate signal differences resulting from auditory stimulus history and (ii) isolate visual processing and its modulation by the auditory background manipulations (Escoffier *et al.,* 2015). In each block, control epochs were defined for each target position as the equivalent duration time window in nontarget trials, which were randomly selected without replacement according to the relative frequency of the target position. This subtraction method was applied to both silence and rhythm blocks to render them comparable. We focused our ERP analysis for measure onsets on the P1 over temporal electrodes (T8, TP8, T7, TP7). This electrode selection diverges from many published studies on auditory processing, which pursued effects over fronto-central channels instead. However, visual inspection of the current ERPs revealed the best defined and largest sound-related deflections over temporal cortex. We refer the interested reader to the Supplementary Materials which shows ERPs time-locked to measure onset for all recording channels and includes a statistical analysis of fronto-central effects. Following up on previous work (Escoffier *et al.,* 2015), we explored the target onset N1 and P3 over occipital electrodes (O1, Oz, O2). Analysis time windows were set based on prior research and visual inspection of grand mean voltages and were 110–130 ms, 150–170 ms and 250–400 ms for auditory P1 and visual N1 and P3, respectively.

For the analysis of EEG oscillations, preprocessing was done as described for the ERP with the exception that the data were split into 30 s long epochs after the 0.1 Hz high-pass filtering. The cleaned EEG data were transformed into frequency domain using fast Fourier transform (FFT), resulting in frequency spectrum amplitudes in μV ranging from 0 to 250 Hz with a frequency resolution of 0.033 Hz. To improve signal-to-noise ratio, the frequency spectra were averaged across epochs in each condition and baseline corrected by subtracting from the amplitude at each frequency bin the average amplitude at surrounding frequencies (−0.0667 to −0.1333 Hz to the left and +0.0667 to +0.1333 Hz to the right of each bin) (Lenc *et al.,* 2018). Of interest to us were frequencies corresponding to measure onset (i.e. meter frequency) at 1/3 Hz and the beat position at 4/3 Hz. Amplitudes at these frequencies were analyzed for the same set of electrodes as selected for the ERP. Please note that our electrode selection differs from previous work because we used a reference-free CSD transformation as to better localize/isolate region-specific activity over primary auditory and visual cortex (Kayser and Tenke, 2015). Because of salient differences in the trial numbers left after artifact removal, we matched trial numbers across the factors/levels for the below statistical analyses. Specifically, for each participant we identified the condition with the lowest number of trials and randomly selected trials from the other conditions. Trial numbers matched across conditions averaged to 10.6 with a between subject range of 5–16.

To explore the possibility that 1/3 and 4/3 Hz rhythms were tagged in the EEG, we adopted a previously developed approach that aims at comparing the EEG signal to a processed sound signal modeling cochlear firing patterns (Lenc *et al.,* 2018, 2019b). To this end, we first down-sampled the experimental sound recording for each participant and condition to 5000 Hz. We then applied a Patterson–Holdsworth ERB filter bank with 128 channels to simulate the cochlear filtering (Patterson and Holdsworth, 1996) and entered the result to the Meddis hair–cell model implemented in the Auditory Toolbox in MATLAB (Slaney, 1998). To facilitate comparison with the EEG, the model output was further down-sampled to 500 Hz and then Hilbert transformed as to get the estimated cochlear response envelope. The result was subjected to a fast Fourier transform with a frequency resolution of 0.033 Hz. The amplitudes in the frequency domain were then averaged across the 128 cochlear channels. As for the EEG, the averaged amplitude in each frequency bin was corrected by subtracting the averaged amplitude of its surrounding frequencies.

For statistical analysis, EEG and cochlear model FFT results were separately normalized. To this end, we computed the amplitude mean and s.d. for 1/3 Hz, and this frequency’s harmonics up to the 30th harmonic. We then subtracted from the amplitude of each target frequency (i.e. 1/3 and 4/3 Hz) the mean amplitude and divided the result by the s.d. This process was applied to all experimental sound recordings presented in each condition and for each participant.

All statistical analyses were conducted in R (R Core Team, 2015) with the ez package (Lawrence, 2016). Follow-up analyses of significant interactions were corrected for multiple comparisons using the ‘Bonferroni’ setting with the p.adjust function. As an effect size measure, we report the generalized eta squared (η_G_^2^).

## Results

### Behavioral results

We computed a *d*′ measure based on the normalized probability of false alarms and hits as well as the mean reaction times for hits. Both measures were subjected to separate ANOVAs for the initial silence, the sound blocks and the post-sound silence blocks. Time (on-beat, off-nonmetrical, off-metrical) served as the single repeated measures factor when analyzing the initial silence. Metricality (high, low) and regularity (high, low) were additional factors when analyzing the sound blocks. During the post-sound silence, only metricality and regularity were explored due to an overall reduction in trial numbers.

The *d*′ scores ranged across participants and conditions from 1.53 to 4.26 and were overall very high, implying good performance. An ANOVA with *d*′ as the dependent variable revealed no effect for the initial silence block (*P* = 0.576), only a marginal three-way interaction during the sound blocks (*F*[2,126] = 2.64, *P* = 0.075, η_G_^2^ = 0.003) and nonsignificant effects during the post-sound silence blocks (all *P* > 0.168). Here and elsewhere, analysis of post-sound silence blocks was done to explore the possibility of a carry-over effect from the sound blocks.

**Fig. 4:**
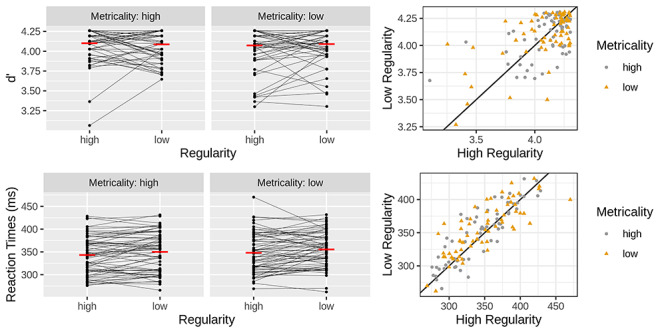
Behavioral results for the sound blocks. The upper row shows *d*′ data and the lower row reaction time data. Individual black dots illustrate individual participants. In the left graph, the thin black lines connecting dots link the two conditions for a given participant. The red line represents the condition mean. The right graph contrasts the two levels of regularity as a function of metricality. It presents the same data as shown in the left graph. Points falling above the black diagonal represent participants with greater values (higher *d*′ and longer RTs) in the low as compared to the high regular condition. Effects were nonsignificant for *d*′. Reaction times were faster when background regularity and metricality were high as compared to low.

For RTs, there was again no effect for the initial silence (*P* = 0.645). For the sound blocks, the main and interaction effects of time were nonsignificant (all *P* > 0.644). However, we found faster responses when metricality was high as compared with low (*F*[1,63] = 6.27, *P* = 0.015, η_G_^2^ = 0.003) and when regularity was high as compared with low (*F*[1,63] = 8.11, *P* = 0.006, η_G_^2^ = 0.007). Notably, the regularity effect size was twice that of the metricality effect. For the post-sound silence, effects were again nonsignificant (all *P* > 0.271) (Figure 4).

Last, we conducted a planned comparison testing, specifically the RT entrainment effect observed in previous work (Escoffier *et al.,* 2010, 2015; Brochard *et al.,* 2013). However, rather than comparing on *vs* off-beat target positions, here and elsewhere in the manuscript, we focused on the metricality effect for on-beat targets. Specifically, we compared responses to on-beat targets presented with a high as compared to a low metricality background reasoning that the former should facilitate processing relative to the latter. Contrasting backgrounds is preferable over contrasting target positions because the foreperiod remains constant. To further increase comparability with previous research, we isolated the regular background condition for our analysis. A paired one-sided *t*-test comparing high with low metricality was significant (*t*(63) = 1.76, *P* = 0.041) indicating that a metrical background rhythm facilitated responses to on-beat stimuli, thus replicating previous results. Notably, however, there was a similar tendency for targets occurring at an off-metrical (*t*(63) = 1.44, *P* = 0.077) and an off-nonmetrical position (*t*(63) = 1.4, *P* = 0.083). These results concur with the metricality main we observed in the absence of a metricality by time interaction (*F*[2126] = 0.43, *P* = 0.649) in the primary analysis and conflict with the notion that visual attentional peaks align to the beat position of the auditory background.

### Event-related potentials

**Fig. 5:**
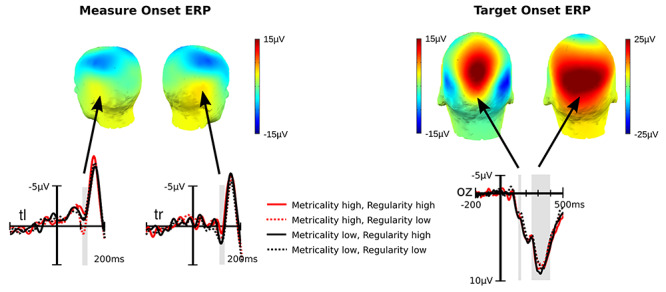
Sound block mean ERP voltages. Maps illustrate the mean amplitude topography for P1 elicited at measure onsets and N1/P3 elicited at target onsets. Temporal electrodes are shown for the first note in each measure and occipital electrodes for the targets occurring at and around the silent beat. For the latter ERP, we show a difference wave derived by subtracting visual target trials from nontarget trials as to remove the influence of preceding or following sounds. Marked in gray are the time windows used to quantify the P1 for measure onset and the N1/P3 for target onsets. There were no significant effects for the auditory P1. The visual N1 was smaller with high as compared to low background regularity. An opposite effect approached significance for the P3.

We derived ERPs time-locked to measure and target onset as to explore meter and beat entrainment, respectively. Both are illustrated in Figure 5.

The first note of each measure was identical with differences emerging only after 131.25 ms, thus giving us enough time to examine the P1 (110–130 ms) without stimulus confounds. The mean P1 amplitudes over temporal electrodes (T8, TP8, T7, TP7) were subjected to an ANOVA with the repeated measures factors metricality, regularity and hemisphere. This revealed a marginal effect of regularity (*F*[1,63] = 2.99, *P* = 0.089, η_G_^2^ = 0.002) and a significant interaction of metricality and hemisphere (*F*[1,63] = 5.79, *P* = 0.019, η_G_^2^ = 0.003). However, follow-up of the latter effect yielded only a marginal P1 reduction when metricality was high as compared to low over the right (*F*[1,63] = 4.42, p_B_ = 0.079, η_G_^2^ = 0.006) an no effect over the left temporal lobe (*F*[1,63] = 0.844, p_B_ = 0.723, η_G_^2^ = 0.001). All other effects were nonsignificant (all *P* > 0.105).

To analyze the target ERP, we identified the mean amplitudes for N1 (150–170 ms) and P3 (250–400 ms) as measured over occipital electrodes (O1, Oz, O2) and subjected them to separate ANOVAs. Due to low trial numbers, we did not pursue the initial silence. For the sound blocks, we conducted an ANOVA with time, metricality and regularity as repeated measure factors and found that when regularity was high, both N1 (*F*[1,63] = 5.89, *P* = 0.018, η_G_^2^ = 0.004) and, but marginally, P3 (*F*[1,63] = 3.82, *P* = 0.055, η_G_^2^ = 0.003) had more positive amplitudes than when it was low. All other effects were nonsignificant (all *P* > 0.255). ERP voltages for the post-sound silence were subjected to an ANOVA with metricality and regularity as repeated measures factors, and the results were nonsignificant (all *P* > 0.262).

Last, we conducted a planned comparison testing specifically the N1 entrainment effect observed previously (Escoffier *et al.,* 2015). To this end, we used a paired one-sided *t*-test to compare high with low metricality for the N1 amplitudes measured over the right hemisphere (O2) in the highly regular on-beat condition and found no condition difference (*P* = 0.278).

### Frequency tagging analysis

We pursued the EEG frequency domain to determine whether sound-related frequencies were significantly amplified in the EEG over auditory and, possibly, visual cortex (Nozaradan *et al.,* 2011, 2018; Lenc *et al.,* 2018). Although there may be some overlap between ERPs and the EEG frequency spectrum, the former highlight event-related information, whereas the latter highlight both event-related and event-unrelated oscillatory activity at frequencies of interest. For the present purpose, we subtracted the normalized cochlear output from the normalized temporal and occipital EEG. A positive score denotes EEG activity exceeding that of the stimulus-driven auditory response model and is thought to index cortical amplification or tagging of cognitively relevant stimulus frequencies. Scores were subjected to two sets of analyses done on sound block data only.

The first set of analyses entailed a *t*-test conducted for each region (temporal, occipital) and frequency (1/3 and 4/3 Hz) on *Z* score differences between EEG and cochlear output averaged across metricality and regularity. This demonstrated that across conditions, measure and beat frequency were significantly amplified in the EEG (all *P*_B_ < 0.004).

Next, we conducted for each region separate ANOVAs with frequency (1/3 and 4/3), metricality and regularity as repeated measure factors. For the temporal region, effects were nonsignificant (all *P* > 0.187). Analysis of the occipital region revealed a significant effect of frequency (*F*[1,63] = 11.65, *P* = 0.001, η_G_^2^ = 0.068) and significant interactions of regularity and frequency (*F*[1,63] = 11.51, *P* = 0.001, η_G_^2^ = 0.018) and of metricality, regularity and frequency (*F*[1,63] = 4.16, *P* = 0.045, η_G_^2^ = 0.006). The interaction of metricality and frequency was marginally significant (*F*[1,63] = 3.69, *P* = 0.059, η_G_^2^ = 0.006), and all other effects were nonsignificant (all *P* > 0.25).

**Fig. 6:**
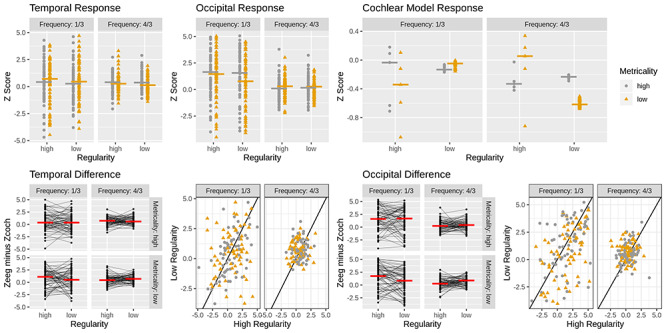
*Z*-scored EEG and cochlear model response to the sound backgrounds. Upper row graphs show the individual *Z*-scores for the temporal and occipital EEG (left) and the cochlear model (right). Lower row graphs show the difference score obtained when subtracting cochlear from temporal (left) and occipital EEG *z*-scores (right). Dots/diamonds represent individual participants. The red vertical lines represent the condition mean. The within-participant regularity effects are represented by the thin lines in the lower left graph and by data point distance from the diagonal in the lower right graph. Statistical analyses yielded only nonsignificant effects for the temporal leads. For occipital leads, there was a significant effect of regularity at both measure onset and beat frequency.

We pursued the significant three-way interaction for each level of frequency. For the measure onset frequency, we observed a significant effect of regularity (*F*[1,63] = 6.87, *P*_B_ = 0.022, η_G_^2^ = 0.014), indicating that EEG amplification was greater for the more regular background. The metricality effect and the interaction of metricality and regularity were nonsignificant (all *P*_B_ > 0.232). For the beat frequency, we again found an effect of regularity (*F*[1,63] = 6.24, *P*_B_ = 0.03, η_G_^2^ = 0.021). However, unlike for the measure onset, low regularity was associated with greater EEG amplification than high regularity. Again, the metricality main effect and the interaction of metricality and regularity were nonsignificant (all *P*_B_ > 0.36) (Figure 6).

## Discussion

The idea that, like motor processes, mental processes have a periodicity that can be aligned or entrained by the metrical structure of external rhythms has gained much popularity in the past few decades (Calderone *et al.,* 2014; Schirmer *et al.,* 2016b; Lakatos *et al.,* 2019; Obleser and Kayser, 2019; Hoehl *et al.*, 2020). Our study fails to support this notion and instead highlights the importance of temporal regularity irrespective of metricality as an optimizer for cognitive and brain functioning. However, before discussing our results in detail, we shortly revisit a few important concepts.

As mentioned in the introduction, metricality is defined by an integer ratio between intervals within a sequence (Figure 1) (Jones, 1976; Grahn and Brett, 2007). It gives rise to feeling a rhythm with an equidistantly spaced perceptual emphasis—the beat—that one can readily tap to. By contrast, non-integer ratios are thought to characterize nonmetrical sequences. Such sequences are not perceived as rhythmical and when asked to tap, one varies, unable to identify a clear beat. Note, however, that the perception of metricality may not be a neatly binary but a continuous phenomenon.

The term regularity refers to whether intervals within a sequence are repeated and/or occur with a consistent order. Like metricality, regularity may be considered on a continuum whereby looping a single interval produces an isochronous sequence that maximizes regularity. Importantly, such an extreme case also maximizes metricality, beat finding and associated tapping performance. Past research on entrainment typically compared an isochronous with a nonisochronous and nonmetrical signal (Miller *et al.,* 2013) or an isochronous signal followed by an isochronously aligned as compared to misaligned target (Jones *et al.,* 2002).

### Does the brain entrain?

In a simple tapping experiment, we established that our stimuli elicited synchronized tapping with the beat when metricality was high and variable tapping when metricality was low. Thus, we replicated previously documented effects of rhythm on motor entrainment (Grahn and Brett, 2007). We then explored auditory and visual stimulus processes to determine whether, they too, had been subject to entrainment and aligned with the beat.

Looking at auditory processes, we asked whether metricality facilitates the representation of background sounds. One relevant variable was the P1 amplitude to the first sound of each background measure. The P1 is traditionally considered an index of perceptual processes and is known to vary as a function of stimulus and mental state characteristics. In this context, smaller amplitudes are associated with reduced attention or processing effort (Luck, 2005; Giuliano *et al.,* 2014). In line with the notion that metricality facilitates the processing of metrically aligned sounds, we observed smaller P1 amplitudes in the high as compared to the low metricality condition over the right temporal region. Notably, however, this effect was small and statistically nonsignificant despite our well-powered sample.

A second variable of interest was the amplitude of meter-relevant frequencies in the EEG thought to index a frequency tagging response (Nozaradan *et al.,* 2011, 2018; Ding *et al.,* 2016; Lenc *et al.,* 2018). This variable was first used to examine the visual perception of stimuli flickering at a certain frequency and in this context is known as a steady-state visual evoked potential (Norcia *et al.,* 2015; Wieser *et al.,* 2016). Its extension to auditory rhythm perception has been much debated (Henry *et al.,* 2017; Rajendran and Schnupp, 2019). As with the present study, relevant stimuli typically vary not only in one frequency. Their temporal envelope and associated frequency representations are significantly more complex creating challenges for interpreting resulting FFT spectra and for linking them between stimulus and EEG (Rajendran and Schnupp, 2019). Additionally, the FFT approach discards temporal information, leaving uncertain whether and in what way meter- and beat-related frequency representations in stimulus and EEG may vary at different moments in time.

Despite these problems, however, the frequency tagging response is interesting (Lenc *et al.,* 2019a) and in conjunction with other measures elucidates the relation between external and internal oscillations. Notably, in our hands, frequency tagging indicated that important stimulus frequencies are amplified in the EEG over the temporal region, which given we applied a CSD transformation, may index activity of underlying auditory cortex (Kayser and Tenke, 2015). Yet although on average both measure onset and beat frequency were amplified above and beyond the auditory stimulus, this amplification or tagging response did not differ between conditions. We suspect that because the measure onset frequency was present consistently irrespective of background and the beat frequency was the fourth measure onset frequency harmonic, the former sufficed to induce a strong frequency tagging response at both frequencies. Importantly, within-measure metricality was irrelevant.

In addition to looking at auditory processing, we also examined visual processing as to elucidate cross-modal entrainment effects. Yet, such effects failed to materialize. Contrary to previous reports, there was no evidence for metricality modulating visual ERPs (Escoffier *et al.,* 2015) or an occipital frequency tagging response. Although, the present study could replicate previous behavioral effects, it showed that they may have sprang from local rather than global temporal processes. Specifically, we observed faster RTs to on-beat visual targets presented on a background with high as compared to low metricality and a control experiment (Supplementary Materials) with blocked foreperiods replicated faster RTs to on-beat as compared with off-beat targets on a background with high metricality (Escoffier *et al.,* 2010; Brochard *et al.,* 2013; Miller *et al.,* 2013). However, the wider context of the collected data revealed that these effects were not rhythmical in nature (for further information on present foreperiod effects see Supplementary Materials). Indeed, a metricality main effect in the absence of an interaction between metricality and target position indicated that high as compared with low metrical backgrounds facilitated the processing of visual targets irrespective of when they occurred.

Together, the present auditory and visual results offer little, if any support for the notion that metricality entrains unimodal and cross-modal perceptual processes. Metricality, if relevant at all, shows complex effects that interact with regularity and that like the regularity effects discussed below may be best understood as facilitating the overall representation of a rhythmic or temporally regular input rather than attention at metrical moments in time.

### Temporal regularity underpins dynamic attending

If, contrary to popular belief, metricality matters little for the temporal dynamics of mental processes, what other stimulus feature could be relevant? Based on previous research as well as the current results, we suggest a role for temporal regularity (for a more extended discussion see Hoehl *et al.*, 2020).

Evidence for the brain’s ability to leverage on temporal regularity comes from three separate literatures. First, relevant work has used the mismatch negativity (MMN), a negative deflection in the ERP elicited to rare deviant sounds presented among frequent standards in a task-irrelevant and unattended stimulus stream. The MMN shows for simple physical as well as higher-order rule-based deviations (for reviews see Näätänen *et al.,* 2010; Paavilainen, 2013) including those arising from temporal stimulus properties (Yabe *et al.,* 1997; Tse and Penney, 2006; Schirmer *et al.,* 2016a). Thus, it seems the brain automatically represents input and derives regularities against which further input is processed. Temporal regularities are just one dimension of this.

A second line of evidence comes from a phenomenon called statistical learning. It was first discovered in infants in a word-learning experiment (Saffran *et al.,* 1996). Four three-syllable pseudowords were presented in random order such that the transition probabilities of syllables within words were high, whereas those of syllables between words were low. After only 2 min of exposure, infants picked up on these probabilities and could use them to segment continuous speech into individual words. This finding has been replicated across different ages, senses and stimulus features (for a review see Fiser and Lengyel, 2019). Additionally, a recent ERP study manipulating transition probabilities in a sequence of tone triplets found overlap in the processes supporting statistical learning and generating the MMN (Mittag *et al.,* 2016).

Last, evidence for the relevance of regularity in guiding the temporal course of mental processes comes from work by Breska and Deouell (2017), probing rhythmic entrainment using a paradigm similar to that of Jones *et al.* (2002). A visual stimulus sequence preceded a visual target. The sequence was (i) isochronous in that a single interval was looped, (ii) nonisochronous in that a single interval was repeated with varying, nonmetrical delays or (iii) was random, comprising varying intervals and delays with non-integer ratios. Compared to the random condition, the other conditions elicited greater delta phase coherence in the EEG and faster response times when subsequent targets occurred with the sequence-specific interval as opposed to a faster/slower interval. Moreover, both effects were related as delta phase at target onset predicted reactions times, and they existed irrespectively of whether the sequences were isochronous (for a critique see Obleser *et al.,* 2017).

The present data corroborate and extend this work by examining metricality and regularity orthogonally, dissociating rhythmic from broader temporal expectation effects and pursuing cross-modal entrainment. Across our different measures, regularity seemed more important than metricality. Regularity marginally reduced the auditory P1 elicited at measure onset. Additionally, it modulated our indexes of visual target processing in a number of ways. First, targets occurring on a high as compared with a low regularity background elicited a smaller N1, a marginally larger P3 and faster reaction times. A smaller N1 has been previously associated with increased predictability or perceptual fluency (Robinson *et al.,* 2018), whereas a larger P3 and other late positivities have been linked to a number of functions including task relevance (Squires *et al.,* 1977; Johnson Jr and Donchin, 1978) and emotional salience (Hajcak *et al.,* 2010; Liu *et al.,* 2016; Schirmer and Gunter, 2017). Regularity also modulated the frequency tagging response over occipital cortex. Tagging was greater for temporally regular as compared with irregular backgrounds for the measure onset frequency, whereas a reversed effect showed for the beat frequency. This suggests that regularity enhanced the representation of the most regular interval across all sound sequences and reduced the representation of that interval’s fourth harmonic possibly because this harmonic offered little information about the timing of individual events in the sound background and events in the foreground visual task.

Taken together, sound background regularity had nonsignificant effects on auditory and small (η_G_^2^ < 0.021), but consistent, effects on visual processing. The nonsignificant auditory influence may be due to the regularity of measure onsets irrespective of condition providing a salient temporal cue that prevented us from observing more subtle responses as a function of condition. In line with this, there was a robust frequency tagging response at both measure onset and beat frequency over temporal leads with all auditory backgrounds. The emergence of a significant visual influence may arise from multisensory integration mechanisms that dynamically link object information distributed across different modalities. Specifically, we speculate that temporally regular sounds facilitate sound representations in uni- and multimodal processing systems, which then benefits visual perception by freeing mental capacity.

### The (limited) power of meter

Given the power of music, the convincing argumentation of DAT and existing empirical data, the present failure to establish robust mental entrainment came as a surprise. The metricality of a rhythm clearly influences us in a special way; how then can this influence be understood?

Perhaps we should think of metricality as a special form of regularity. The integer ratio of sound intervals facilitates interval processing, and the recurrence of certain intervals and interval harmonics enables the perception of a highly regular beat. Moreover, because of the special interval relationships and the regular beat, other less regular elements (e.g. varying interval durations, small deviations from the beat) may be more readily tolerated and integrated into ongoing sensory processing.

As such, metricality, like other forms of regularity, makes stimulus encoding more efficient and frees resources for the processing of other information. Importantly, the present study shows this latter effect is independent of whether such information aligns with the beat. Additionally, metricality plays a highly significant and likely specific role in synchronizing motor output. Although here we found little support for rhythmically aligned attending, we observed a strong role of meter in rhythmically aligned tapping. Similarly, work by Breska and Deouell (2017) found that isochronous timing specifically increased motor-preparatory but not other activity in the brain. Thus, instead of broadly entraining the mind, metrical rhythms may be more relevant in organizing behavior. In this manner, they may usefully facilitate music making, dancing and other social activities that humans perform in smaller and larger groups (Launay *et al.,* 2016).

### Caveats and directions for future research

Because the present focus was on rhythmic entrainment, our visual target time points were either metrically aligned or misaligned with an auditory background stimulus. Moreover, they did not vary based on the actual intervals present in the background stimulus. As such we were unable to test whether intervals irrespective of their metricality modulate perceptual processes dynamically by enhancing responses at interval congruous and/or impairing responses at interval incongruous time points. Such a test was first attempted by Breska and colleagues (2017) and presents an important direction for future research.

Additionally, although the present study showed measure convergence in the relevance of background metricality and regularity for auditory and visual processing, it failed to establish links between the different measures. As such it cannot speak to how frequency tagging facilitates early ERPs or how both are relevant in explaining the speed and accuracy of visual target detection. We have attempted this at the subject level without obtaining meaningful results. Likely one must adopt a trial-based within-subject approach to shed light on measure relationships and to develop a framework for the mechanisms by which temporal interval and metrical properties organize mental processes.

## Conclusions

The idea that external rhythms entrain the peaks and troughs of internal processes like visual attention has been very popular and attracted substantial scientific inquiry (for reviews see Calderone *et al.,* 2014; Schirmer *et al.,* 2016b; Lakatos *et al.,* 2019; Obleser and Kayser, 2019; Hoehl *et al.*, 2020). Yet, whether any temporal alignment is indeed rhythmical in that it springs from and maps onto an underlying meter has not been clearly established. The present findings contradict this possibility and instead suggest that the timing of internal processes depends on external stimulus regularities independently of their metricality. Temporal regularity rather than metricality emerged as a fundamental principle for mental resource allocation. Although unexpected on the backdrop of current entrainment theory, our findings converge with research on the role of repetition and prediction in optimizing human functioning (Kilner *et al.,* 2007; Friston, 2010).

## Conflict of interest

The authors declare no conflict of interest.

## Funding

This work was supported by a GRF grant awarded by the Hong Kong Research Grants Council to Annett Schirmer (14612318).

## Supplementary Material

nsaa077_Supp
