# Supplementary material for: Musical rhythm effects on visual attention are non-rhythmical: evidence against metrical entrainment
Source: Soc Cogn Affect Neurosci. 2020 Jun 8;16(1-2):58–71. doi: 10.1093/scan/nsaa077 (PMC7812633; doi:10.1093/scan/nsaa077)
Supplement: nsaa077_Supp [file nsaa077_supp.zip › File008_nsaa077.pdf]

Supplementary Materials

1) Behavioral Results - Target Position Plotted as a Function of Metricality and Regularity

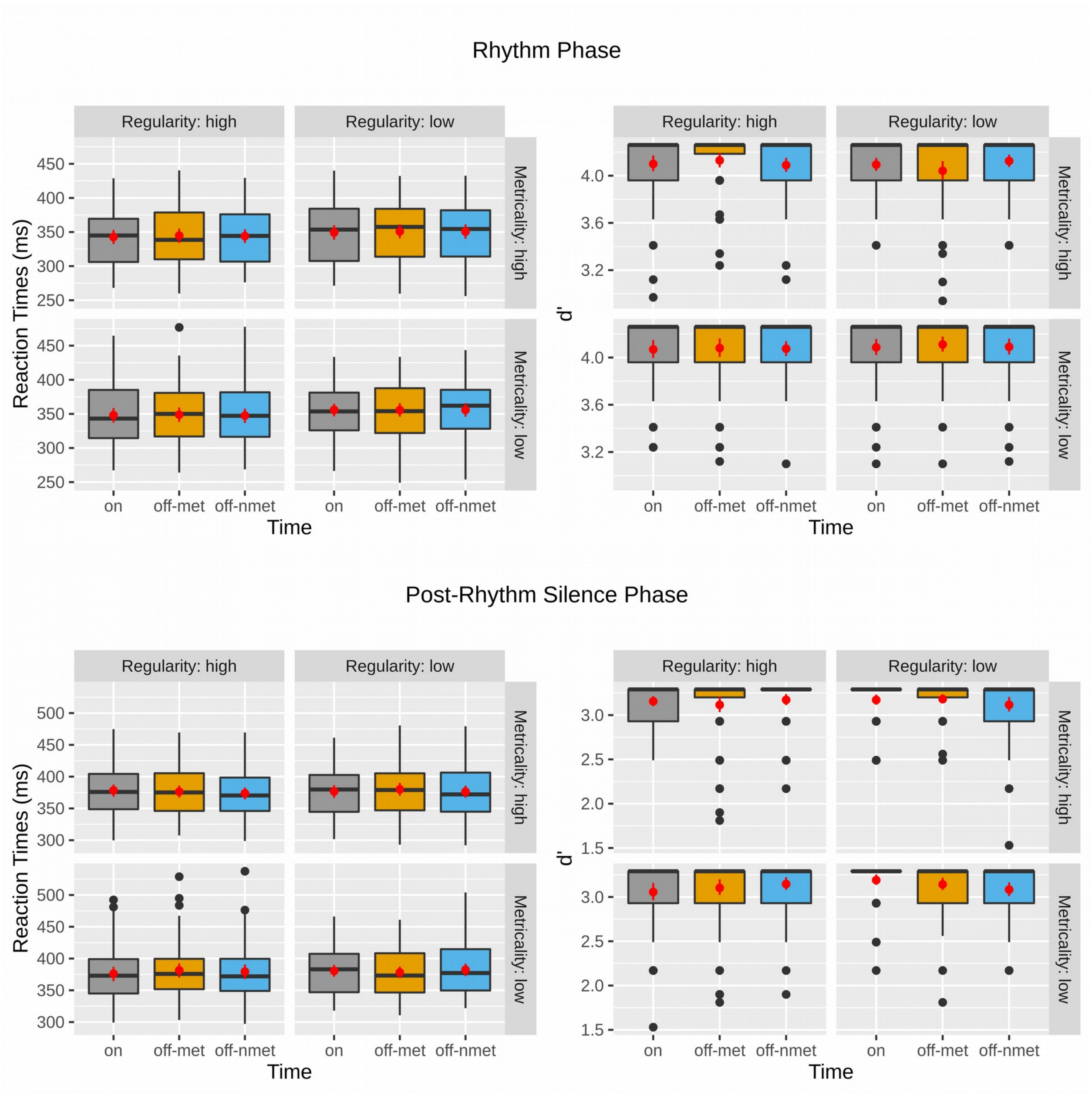

Supplementary Figure 1. Behavioral results. Mean reaction times and d’ scores are shown as box-and-whisker plots overlaid with the mean and its 95% confidence interval in red.

2) Hazard Function and Foreperiod Effects

One issue with studying attentional entrainment is how to control for local temporal effects such as variations in the hazard function or foreperiod. Previously this was attempted by presenting on and off-beat positions such that their foreperiods would facilitate responses to the latter rather than the former (Brochard, Tassin, & Zagar, 2013; Escoffier, Sheng, & Schirmer, 2010). However, apart from the delay between a pre-target sound and the target, many other factors including the distribution of such delays within a study are relevant. Thus, response times must be considered the result of complex interplays between local and global temporal cues.

In the present study, we addressed this issue by varying the metricality of the background rhythm and examining how it modulated responses to on-beat targets and whether a similar effect

was smaller or absent on off-beat targets. Thus, we could isolate the effect of global temporal cues associated with the background rhythm. Additionally, as we varied the timing of on and off-beat targets between two subject groups, we could pursue local timing effects which we will report here.

We examined reaction times (RT) and d' recorded during the rhythm phase of the experiment using an ANOVA with *Metricality*, *Regularity*, and *Time* as repeated measures factors and *Group* as a between subjects factor. We expected Group 1 to shed light on the role of foreperiod distribution. Given that here the on beat position was the temporal mean of all presented positions, on-beat responses might be facilitated because their local temporal representations forms a prototype (i.e., the average of all levels of *Time*). Group 2, by contrast, had a target timing distribution in which no single temporal position was at the mean of presented positions. Moreover, the on-beat position was away from the mean and thus less salient. Therefore, we ventured that Group 2 should reveal foreperiod effects that are less dependent of the overall foreperiod distribution. In sum, if actual foreperiod and foreperiod distribution matter, we should observe an effect of Group on the present results.

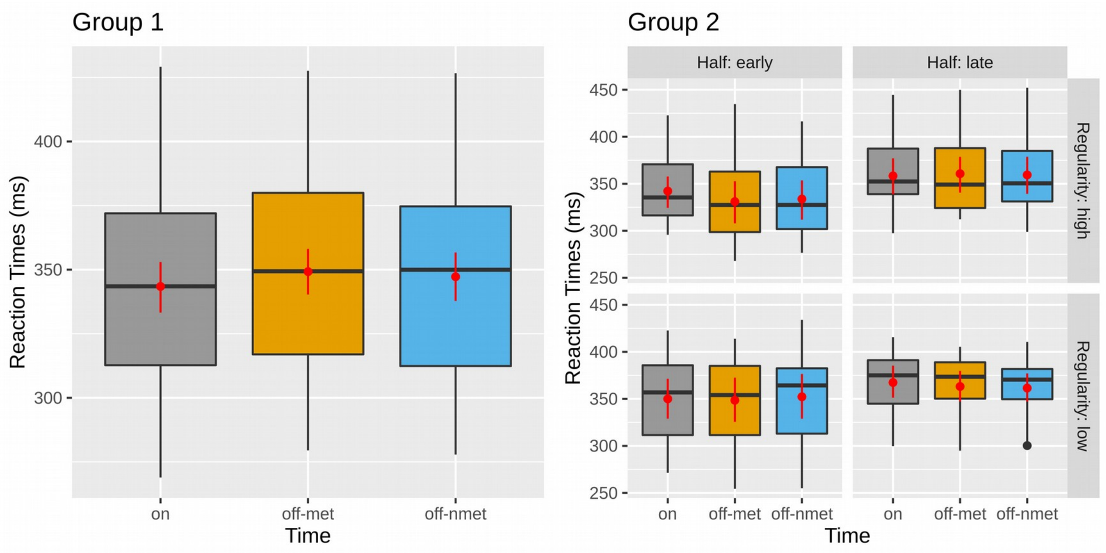

Supplementary Figure 2. Reaction times box-and-whisker plots with the subject mean and confidence interval added in red. Group 1 and Group 2 target timings are illustrated in Figure 3 in the main text.

Our findings are illustrated in Supplementary Figure 2. RT analysis revealed the expected interaction between *Group* and *Time* (F[2,124]=10.53, p<.0001, ges=.002). All other effects involving *Group* were non-significant (ps>.319). Looking at Group 1, there was a main effect of *Time* (F[2,62]=9.08, p=.0003, ges=.003) indicating that on-beat responses were faster than both metrical (F[1,31]=10.58, p=.0003, ges=.005) and nonmetrical off-beat responses (F[1,31]=5.78, p=.022, ges=.002), with the latter two differing only marginally (F[1,31]=3.6, p=.067, ges=.0006). We pursued Group 2, by adding *Half* as another between subject factor indicating whether off-beat positions occurred before or after the on-beat position. The ANOVA revealed a marginal effect of *Time* (F[2,62]=2.86, p=.065, ges=.001) and a significant interaction of *Time*, *Regularity*, and *Half* (F[2,60]=5.14, p=.009, ges=.002). All other effects of *Time* were non-significant (ps>.825).

Looking at individuals exposed to targets occurring before the beat revealed an effect of *Time* (F[2,30]=3.72, p=.036, ges=.003), *Regularity* (F[1,15]=6.16, p=.025, ges=.025), and a *Time* by *Regularity* interaction (F[2,30]=3.23, p=.054, ges=.003). Follow-up analysis suggested that for the regular background condition (F[2,30]=4.35, p=.022, ges=.012), metrical (F[1,15]=5.83, p=.029, ges=.017) and nonmetrical offbeat targets (F[1,15]=3.6, p=.077, ges=.011) elicited faster RTs than on-beat targets. RTs for off-beat targets did not differ (p=.262). The *Time* effect was non-significant for the irregular background condition (p=.323).

Looking at individuals exposed to targets occurring after the beat revealed non-significant results (ps>.163)

Analysis of d' revealed no significant effects of *Group* (ps>.169).

3) Free Tapping and Behavioral/Neuronal Entrainment

We measured the inter-tap-interval recorded from the free tapping task and computed the mean for each participant. We then subtracted 750 ms (our inter-beat-interval of the metrical background rhythms). A one-sample t-test against 0 was significant indicating that, on average, participants tapped significantly slower than the beat of our background rhythms ($t(63)=3.3$, $p=.001$, mean=148, CI: 59 to 238).

Next, we took the absolute value of the above difference score, quantifying the magnitude of deviation of the subject's tapping rate from the stimulus beat, and conducted three Pearson correlation analyses aimed at probing a potential role of a person's spontaneous motor tempo for the predicted entrainment effects – that is (i) the beat-frequency tagging and (ii) a facilitation to on-beat targets as a function of metricality. Dependent measures were the EEG beat frequency amplitude, the visual target N1 over the right hemisphere, and reaction times. For the first measure, we subtracted, for the low regularity condition, the beat amplitude associated with the low from that with the high metrical rhythm. For the latter two measures, we subtracted high from low metricality for on-beat targets presented with a regular rhythm. These three measures quantified the entrainment effect with a larger value correspond to a bigger entrainment effect.

For the EEG beat frequency, a positive relationship ($r=.26$, $p=.039$, CI: .01 to .47) indicated that for individuals whose spontaneous tapping rate was further away from the stimulus beat, power was more strongly amplified by metricality in the low regularity condition. An opposite trend for the N1 was non-significant (on-beat, $r=-.112$, $p=.376$, CI: -.349 to .137; all off-beat, $r=.06$, $p=.65$, CI: -.19 to .3). However, the correlation between the tapping score and RTs produced a significantly negative relationship ($r=-.261$, $p=.037$, CI: -.48 to -.02) indicating that a larger RT entrainment effect was associated with a smaller difference between an individual's spontaneous tapping rate and the experimental rhythms' inter-beat-interval. Although this effect was non-significant for metrical and nonmetrical off-beat positions, coefficients were not significantly different from that in the on-beat condition. Moreover, a correlation of scores averaged across on and off-beat targets revealed a marginal effect ($r=-.24$, $p=.055$, CI: -.46 to -.006).

4) Control Experiment Blocking On- and Off-Beat Foreperiods

Please recall that results differ when foreperiods vary as compared to when they are being held constant within an experimental block (Niemi & Näätänen, 1981). Thus, the lack of evidence for rhythmic entrainment as postulated by DAT may have been due to the event-related presentation of target positions. Therefore, we conducted a control experiment on a separate group of participants using a blocked foreperiod design.

Participants. We examined 32 participants that did not participate in the main experiment. Half of the participants were female aged 19.7 (SD 1.7) and half were male aged 20.9 (SD 3.3). No participants were excluded from data analysis.

Procedure. Instead of a change in fixation cross colour, neutral and happy faces served as targets and appeared left or right of fixation once during each measure in a block. The participants had to indicate the face location by pressing a left or right response button. Faces were presented at the same early off and on-beat positions as for Group 2 in the main experiment. However, rather than occurring randomly throughout a given background block, the different target timing positions were grouped together. Specifically, the four sound blocks were divided into four sub-blocks each such that there was one sub-block for each target timing position. The order of sub-blocks and supra-

blocks was counterbalanced across participants using a Latin square design. Each sub-block consisted of 48 trials, divided equally among all combinations of emotion (happy, neutral) and face location (left, right) which occurred in random order.

Results. As was done previously, we compared, for the high regularity condition, mean correct RTs to on-beat and nonmetrical off-beat targets using a one-sided paired t-test. The result was significant (t=-1.87, df=31, p=.035) indicating that on-beat targets were responded to faster than off-beat targets. As such we could replicate previous results (Brochard et al., 2013; Escoffier, Herrmann, & Schirmer, 2015; Escoffier et al., 2010). Yet, this result could not be clearly confirmed with the better controlled comparison of high vs low metricality for on-beat targets (t=-1.29, df=31, p=.103). Moreover, an ANOVA on the full data set with *Metricality*, *Regularity* and *Time* as repeated measures produced main effects of *Metricality* (F[1,32]=8.9, p=.005, ges=.006) and *Regularity* (F[1,32]=20.92, p<.0001, ges=.024) as well as a *Metricality* by *Regularity* interaction (F[1,32]=4.67, p=.038, ges=.004) indicating that *Metricality* facilitated responses across on and off-beat targets when the background rhythm had low (F[1,32]=11.03, p=.002, ges=.018) but not high regularity (p=.732). Importantly, all other effects were again non-significant (ps>.385).

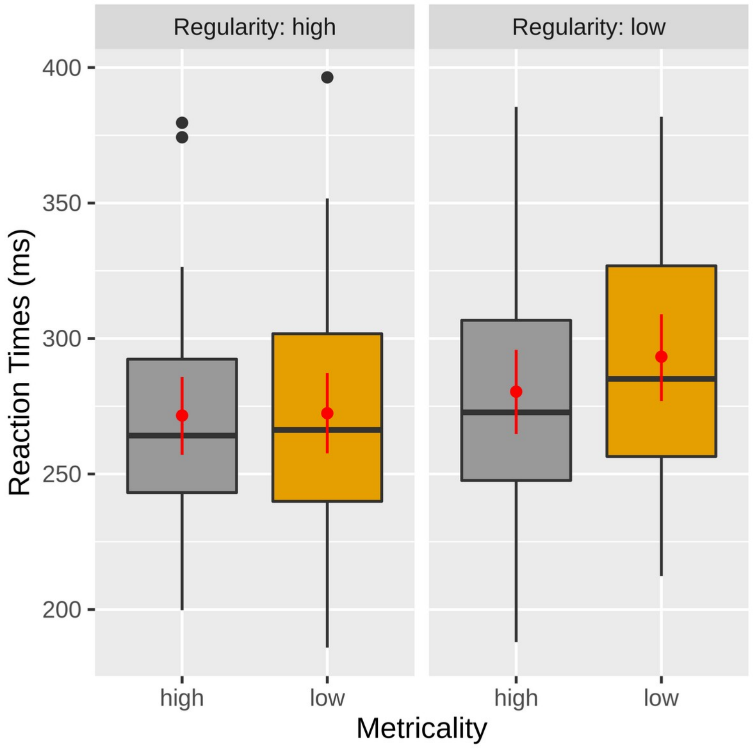

Supplementary Figure 3. Behavioral results from the control experiment. The red overlay represents the the mean of the reaction times and its 95% confidence interval.

5) Visual illustration of measure onset ERPs

The graphs below show the ERPs time-locked to measure onsets for the different background conditions.

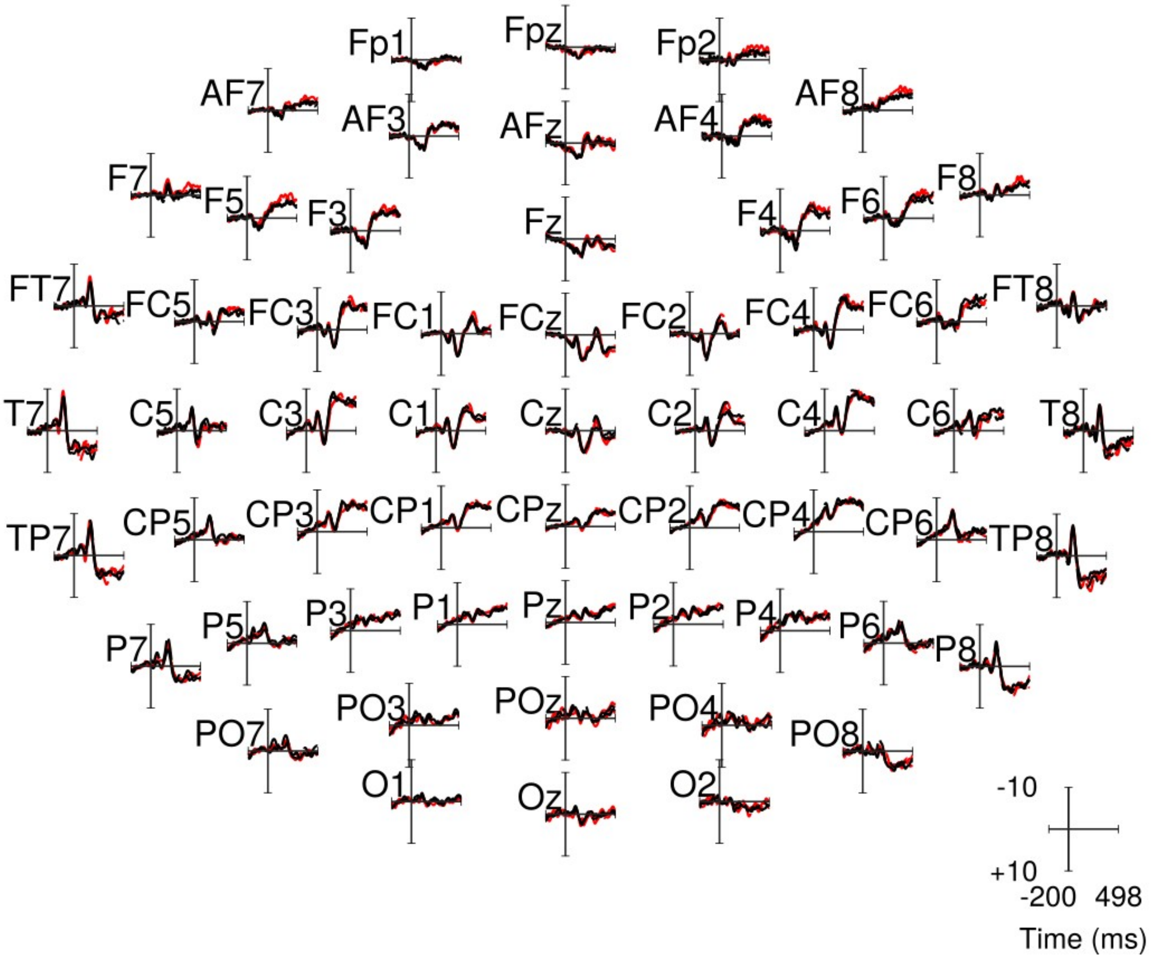

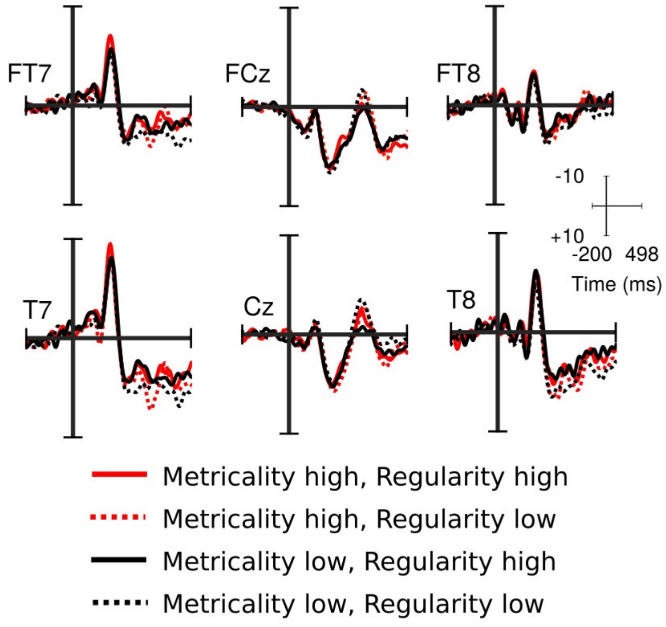

6) Statistical analysis of fronto-central effects

Because in previous work on auditory perception, analysis has been focused on fronto-cental channels, we here provide such an analysis for reference. Specifically, we focus on the mean values obtained from FCz, FC1, and FC2.

Measure Onset ERPs. Visual inspection of fronto-central ERPs revealed an early positive deflection between 60 and 80 ms and a subsequent negative deflection between 100 and 120 ms following stimulus onset. We refer to both as P1 and N1, respectively. Separate ANOVAs on the mean voltages computed across the two time ranges were non-significant for both the P1 ($ps>.19$) and the N1 ($ps>.34$). Last, we also conducted an analysis for the same time window used to analyze the temporal P1. Again the results were non-significant ($ps>.46$).

Frequency Tagging. As was done for temporal and occipital regions as document in the main text, we subjected the difference score between fronto-central and cochlear values to an ANOVA with *Frequency* (1/3 and 4/3), *Metricality*, and *Regularity* as repeated measures factors. This returned a main effect of Frequency ($F[1,63]=6.63$, $p=.012$, $\eta_G^2=.035$) indicating that the measure frequency was more strongly tagged than the beat frequency. Additionally, there was an interaction of Metricality and Regularity ($F[1,63]=4.85$, $p=.031$, $\eta_G^2=.007$). Bonferroni adjusted follow-up comparisons yielded a non-significant effect of Metricality when Regularity was high ($p=.71$), and a marginal effect when Regularity was low ($F[1,63]=5.02$, $p_B=.057$, $\eta_G^2=.016$). The latter reflected a tendency of high metricality to reduce frequency tagging relative to low metricality when regularity was low. All other effects were non-significant ($ps>.165$).
